# Ovarian Metastatic Recurrence of Cervical Squamous Cell Carcinoma: A Case Report

**DOI:** 10.7759/cureus.81932

**Published:** 2025-04-09

**Authors:** Imane Slimani, Fatima Safini, Achraf Miry, Sanae Abbaoui, Bouchra Amaoui

**Affiliations:** 1 Radiation Therapy, Souss-Massa University Hospital, Agadir, MAR; 2 Radiation Therapy, Faculty of Medicine and Pharmacy, Ibn Zohr University, Agadir, MAR; 3 Biomed Laboratory, Faculty of Medicine and Pharmacy, Ibn Zohr University, Agadir, MAR; 4 Pathology, Souss-Massa University Hospital, Agadir, MAR; 5 Pathology, Faculty of Medicine and Pharmacy, Ibn Zohr University, Agadir, MAR; 6 Pathology, Souss-Massa University Hospital, Medical School of Agadir, Agadir, MAR; 7 Biomed Laboatory, Faculty of Medicine and Pharmacy, Ibn Zohr University, Agadir, MAR

**Keywords:** cervical cancer, concomitant chemo-radiotherapy, locally advanced cervical cancer, ovary metastasis, recurrent

## Abstract

Ovarian metastases from squamous cell carcinoma of the uterine cervix are extremely rare. We present the case of a 57-year-old patient who was followed for stage IVA cervical cancer according to the International Federation of Gynecology and Obstetrics (FIGO), initially treated with concurrent chemoradiation. Two years after complete remission, she developed bilateral ovarian metastases, which were incidentally discovered and confirmed by histopathological and immunohistochemical analysis. This case represents the first description of ovarian metastases following irradiation of a locally advanced cervical cancer. The occurrence of ovarian metastases in this context, where the ovaries had been irradiated, raises questions about tumor biology and treatment resistance.

## Introduction

Cervical cancer is one of the most common cancers in women worldwide, ranking eighth in terms of cancer locations and accounting for 3.3% of all cancers, with 662,301 new cases globally in 2022 [1]. In August 2020, the World Health Assembly adopted a global strategy to combat cervical cancer, focusing on the prevention, screening, and effective treatment of precancerous lesions as well as invasive cancer [2].

One of the major challenges in cervical cancer is the occurrence of locoregional and metastatic recurrences. According to the International Federation of Gynecology and Obstetrics (FIGO), the recurrence rate ranges from 11% to 22% for stages IB-IIA and from 28% to 64% for stages IIB-IVB [3]. The typical sites of recurrence are the pelvic organs and lymph nodes, with more rare distant metastases, particularly to the liver, adrenal glands, lungs, and bones [4]. Ovarian metastases (OM) from squamous cell carcinoma (SCC) of the uterine cervix are extremely rare, accounting for 1.4% of all metastases [5].

## Case presentation

**Figure 1:**
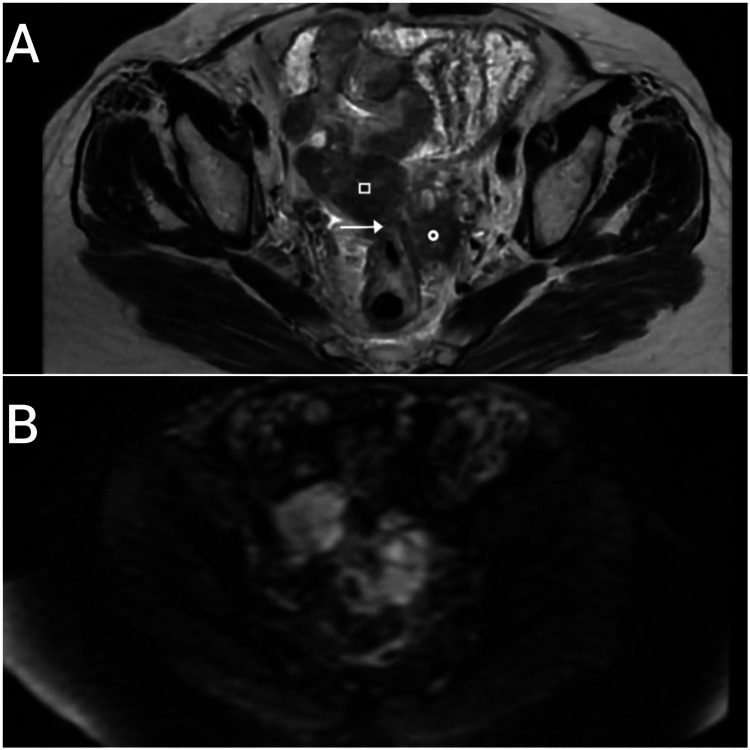
Axial T2 and diffusion of pelvic magnetic resonance imaging (MRI) (A) Axial T2 pelvic MRI: rectosigmoidien invasion (arrow), mass in the right ovary (square), mass in the left ovary (circle). (B) Diffusion pelvic MRI: diffusion restriction in both ovarian masses.

A 57-year-old woman, with no significant medical history, has been under follow-up since 2022 for moderately differentiated SCC of the uterine cervix, staged as FIGO stage IVA. She received external beam radiotherapy of 46 Gy in 23 fractions along with concurrent weekly cisplatin at 40 mg/m², followed by four fractions of intracavitary brachytherapy of 7 Gy each. Pelvic lymphadenopathies with gross involvement were further boosted with an additional dose, bringing the total dose to 66 Gy. Two years after primary treatment, the patient presented for routine surveillance with an abdominopelvic ultrasound, which revealed a left latero-uterine mass measuring 35 × 29 mm and a right latero-uterine lesion measuring 26 × 15 mm. Clinical examination showed no abnormalities. Pelvic magnetic resonance imaging (MRI) (Figure 1) revealed bilateral ovarian masses with a mixed solid-cystic composition, predominantly solid, measuring 45 × 35 mm on the right and 48 × 30 mm on the left. The left ovarian mass was in contact with the lateral wall of the rectosigmoid junction, with no clear demarcation. No pelvic or abdominal lymphadenopathy was noted. Diffusion-weighted imaging demonstrated marked restriction with low adenocarcinoma (ADC) values, suggesting high cellularity. Post-contrast sequences showed intense and rapid enhancement of the solid component. These imaging features, along with the bilateral involvement, support our diagnosis of ovarian metastasis.

A thoracoabdominopelvic CT scan revealed a left latero-uterine solid-cystic mass measuring 60 × 31 × 38 mm, locally infiltrating and compressing the adjacent ureter with moderate ureterohydronephrosis. Additionally, a subcapsular hepatic nodule in segment VII and another left lateral-basal pulmonary nodule of secondary appearance were identified.

**Figure 2:**
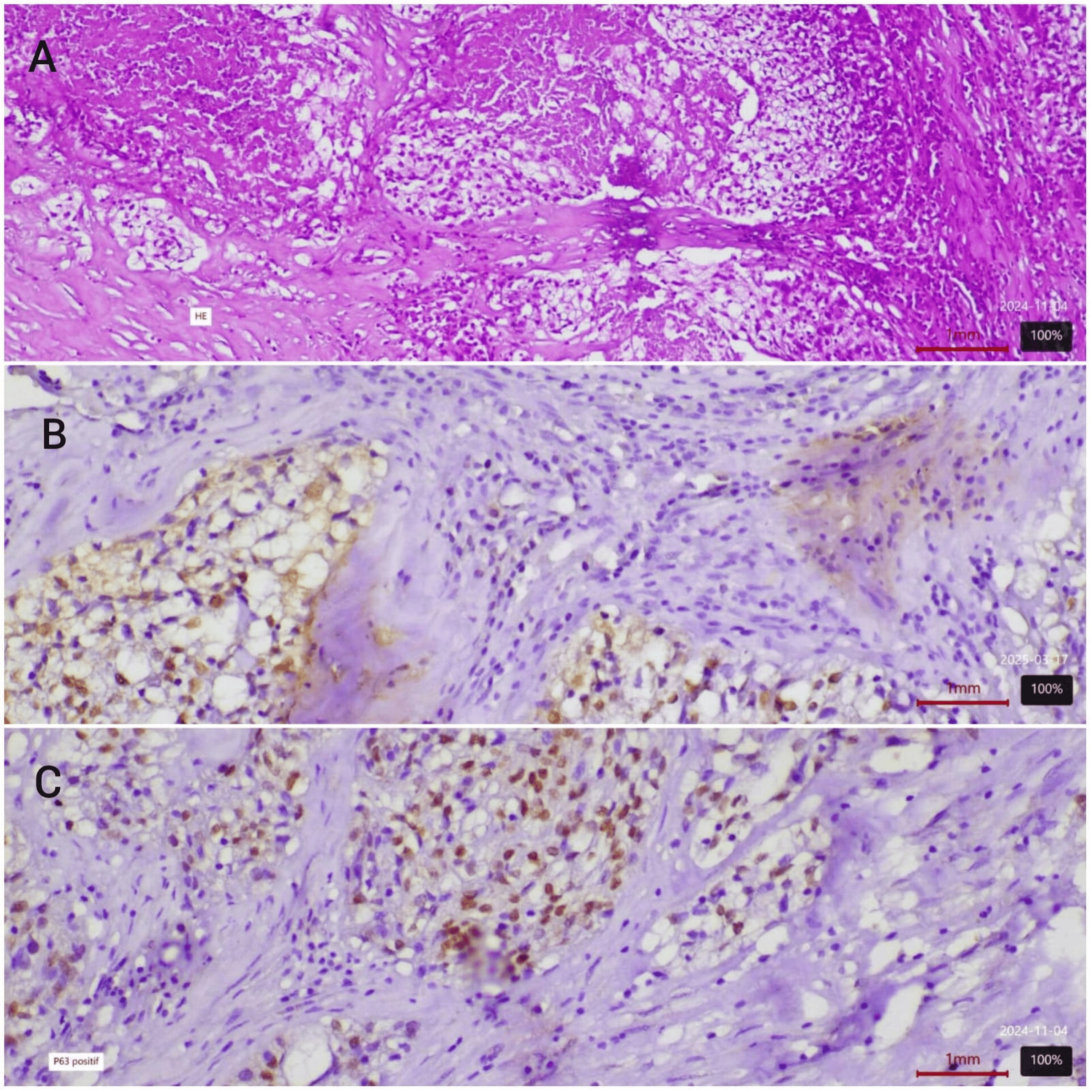
Histopathological image of ovarian tumor (A) Photomicrograph of the ovarian mass revealing a poorly differentiated infiltrating carcinoma (H&E, 100X). (B) Photomicrograph revealing nuclear expression of p16 by neoplastic cells (anti-p16, 200X). (C) Photomicrograph revealing positive nuclear expression of p63 in IHC (anti-p63, 200X).

The patient underwent exploratory surgery, which included a right annexectomy, left ovarian biopsy, and biopsies of the peritoneum and greater omentum. Histopathological examination revealed bilateral ovarian localization of a poorly differentiated infiltrating carcinoma. Biopsies of the greater omentum and peritoneum showed no tumor infiltration, while the ascitic fluid sample revealed carcinoma cells. Immunohistochemical analysis (Figure 2) demonstrated positive expression of P63 (Figure 2B) and CK7, with intense localized expression of P16 (Figure 2C), while PAX8, CDX, and CK20 were negative. This immunophenotypic profile is consistent with a cervical-uterine origin of the lesion.

After multidisciplinary discussion and pretreatment evaluation, the patient was referred to the medical oncology department for systemic treatment. The patient died after six months of medical management.

## Discussion

OM account for 10 to 15% of malignant ovarian tumors, with the majority arising from the genital tract [6]. OM from cervical cancer are rarely reported in the literature, with an overall incidence estimated between 0.9% and 2.2% [7]. Patients with cervical ADC have a higher risk of developing OM compared to those with SCC, with an overall incidence of 3.61% in ADC cases and 1.46% in SCC cases [8]. Cervical cancer can spread to the ovaries either through hematogenous dissemination or via lymphatic and trans-tubal pathways [8,9]. Due to the rich lymphatic system of the ovaries, lymphatic metastases are the most common.

Several risk factors for ovarian metastases in cervical cancer have been reported in the literature, including age (>40 years), tumor size (bulky tumor), pelvic lymph node involvement, vascular emboli, parametrial involvement, uterine body extension, and deep stromal invasion [5]. In the present case, the patient exhibited all of these risk factors.

A review of articles on the rare occurrence of ovarian relapses in cervical cancer revealed five cases involving patients with stage IB cervical cancer, initially treated with surgery (hysterectomy, pelvic lymphadenectomy, and ovarian transposition) without adjuvant therapy, with OM developing 2-10 years after the initial treatment, and the circumstances of OM discovery in the previously reported cases varying from abdominal pain to the palpation of an abdominal mass [9,10-13]. In contrast, in our case, the patient did not report any clinical symptoms, and the metastatic recurrence was incidentally discovered during a routine abdominopelvic ultrasound for surveillance.

To our knowledge, this is the first reported case of bilateral ovarian metastases from locally advanced cervical SCC, initially treated with exclusive concurrent chemoradiotherapy. Due to the patient’s age, menopausal status, and the advanced stage of the disease, she did not receive ovarian preservation strategies, such as ovarian transposition, despite the known benefits of this technique, which preserves ovarian endocrine function in younger women and improves quality of life by preventing severe menopausal symptoms. Ovarian transposition has not been associated with any adverse effects in cases of postoperative radiotherapy, particularly regarding dose distribution, target volume conformity, protection of at-risk organs, and therapeutic efficacy [14,15].

The management of metastatic ovarian recurrences remains challenging due to their rarity. Surgery remains the preferred option when feasible, as in our patient’s case. To enable both accurate diagnosis and effective treatment, a right adnexectomy was performed, along with a biopsy of the left ovary due to tissue alterations observed after radiotherapy.

The KEYNOTE-826 trial demonstrated that the addition of pembrolizumab to cisplatin/paclitaxel chemotherapy, with or without bevacizumab, in patients with PD-L1-positive recurrent cervical cancer significantly improved outcomes. Progression-free survival (PFS) was extended to 10.4 months, and overall survival (OS) at 24 months reached 53% [16].

The addition of bevacizumab to chemotherapy in patients with recurrent, persistent, or metastatic cervical cancer was associated with an improvement of 3.7 months in median OS [17].

## Conclusions

After a literature review, our case represents the first reported instance of metastatic recurrence in the ovaries from stage IVA cervical squamous cell carcinoma (FIGO), treated with concurrent chemoradiotherapy. This case highlights the ability of tumor cells to evade the cytotoxic effects of radiotherapy, even with a significant dose administered to the pelvis. It underscores the importance of rigorous post-treatment surveillance and suggests a re-evaluation of radiotherapy strategies, including the potential inclusion of the ovaries in the target volumes to be irradiated. Additionally, this case prompts further investigation into new therapeutic approaches, such as radiosensitizers or complementary systemic therapies, to improve local control and reduce the risk of recurrence.
